# Delays in Multiple Sclerosis diagnosis (DIMES): protocol for a multicentre, observational study of multiple sclerosis diagnostic pathways in the United Kingdom and Republic of Ireland

**DOI:** 10.1186/s12883-024-03598-z

**Published:** 2024-03-28

**Authors:** Ashvin Kuri, David E. Henshall, Daoud Chaudhry, Setthasorn Zhi Yang Ooi, Qiqi Zhang, Joela Mathews, Alison Thomson, David Rog, Jeremy Hobart, Ruth Dobson, Neurology and Neurosurgery Interest Group (NANSIG)

**Affiliations:** 1 Wolfson Institute of Population Health, Queen Mary University of London, Charterhouse Square, London, EC1M 6BQ UK; 2 Neurology and Neurosurgery Interest Group (NANSIG), London, UK; 3 Deanery of Clinical Sciences, College of Medicine & Veterinary Medicine, The University of Edinburgh, Edinburgh, EH16 4SB UK; 4 Royal Stoke University Hospital, University Hospitals of North Midlands, Stoke-on-Trent, ST4 6QG UK; 5 Cardiff University School of Medicine, Cardiff, CF14 4XN UK; 6 Department of Neurology, Royal London Hospital, London, UK; 7 Department of Neurology, Manchester Centre for Clinical Neurosciences, Salford, Manchester M6 8HD UK; 8 Peninsula Medical School, Faculty of Health, University of Plymouth, Plymouth, PL6 8BT Great Britain

**Keywords:** Multiple Sclerosis, Diagnostic pathways, Healthcare services

## Abstract

**Background:**

Multiple sclerosis (MS) is a leading cause of non-traumatic disability in young adults. Accumulating evidence indicates early diagnosis and early treatment improves long-term outcomes. However, the MS diagnostic pathway is increasingly complex, and delays may occur at several stages. Factors causing delays remain understudied. We aim to quantify the time taken for MS to be diagnosed, and characterise the diagnostic pathway and initial care provided, in the United Kingdom (UK) and Republic of Ireland (ROI).

**Methods:**

Delays In MultiplE Sclerosis diagnosis (DIMES) in the UK and ROI is a multicentre, observational, retrospective study that will be conducted via the Neurology and Neurosurgery Interest Group (NANSIG) collaborative network. Any hospital in the UK and ROI providing an MS diagnostic service is eligible to participate. Data on consecutive individuals newly diagnosed with MS between 1st July 2022 and 31st December 2022 will be collected. The primary outcomes are 1) time from symptoms/signs prompting referral to neurology, to MS diagnosis; and 2) time from referral to neurology for suspected MS, to MS diagnosis. Secondary outcomes include: MS symptoms, referring specialties, investigations performed, neurology appointments, functional status, use of disease modifying treatments, and support at diagnosis including physical activity, and follow up. Demographic characteristics of people newly diagnosed with MS will be summarised, adherence to quality standards summarised as percentages, and time-to-event variables presented with survival curves. Multivariable models will be used to investigate the association of demographic and clinical factors with time to MS diagnosis, as defined in our primary outcomes.

**Discussion:**

DIMES aims to be the largest multicentre study of the MS diagnostic pathway in the UK and ROI. The proposed data collection provides insights that cannot be provided from contemporary registries, and the findings will inform approaches to MS services nationally in the future.

**Supplementary Information:**

The online version contains supplementary material available at 10.1186/s12883-024-03598-z.

## Background

### Background and rationale

Multiple Sclerosis (MS) is a chronic progressive disabling neurological disease. It is a leading cause of non-traumatic disability in young adults worldwide [1].

The therapeutic armamentarium for relapsing remitting MS consists of numerous disease modifying therapies (DMTs). Most DMTs are immunotherapies targeting the dysregulated immune system in MS, and many have shown significant impact on slowing progression and time-to-relapse in clinical trials [2, 3]. Growing evidence suggests early aggressive treatment confers a better long-term prognosis for people living with MS; finding a more concrete answer to this question represents a top 10 research priority area for people with MS according to MS Society UK [4–11].

Crucially, early treatment initiation in MS is predicated upon efficient diagnostic pathways that facilitate early, accurate diagnosis [12]. However, the MS diagnostic pathway is intrinsically complex. Diagnostic confirmation requires the integration of clinical history and examination (to exclude differential diagnoses), magnetic resonance imaging (MRI) findings (to identify MS lesions disseminated in space and time), and potentially laboratory findings (IgG oligoclonal bands in cerebrospinal fluid analysis) [13]. Logistically, the collection and amalgamation of this data in a timely manner poses a challenge. Delays in the diagnostic process will differ between healthcare systems, but may include availability of neurology outpatient appointments, long MRI waiting lists, and access to lumbar puncture services. Furthermore, the impact of the COVID pandemic on services and service recovery has posed additional challenges [14]. In addition to service-level aspects, individual-level factors may play a role, for example whether individuals recognised their initial symptoms merited medical review and/or could relate to MS. Existing literature has identified a number of factors associated with diagnostic delay in MS, including delayed MRI/lumbar puncture [15], nature of presenting symptoms [16–21], lower educational level [17, 19, 20], older age [18, 22, 23], greater number of relapses before diagnosis [18, 22], previous misdiagnosis [18], primary progressive MS subtype [18, 19, 21], male sex [19, 24], greater distance to MS centre [19], increased comorbidity burden [25], negative family history [26], lack of awareness of MS symptoms amongst the general public or healthcare professionals (non-MS specialist diagnosing doctor) [27], and lack of familiarity with the McDonald criteria [16, 27, 28]. There is a need to evaluate and assess such delays in the UK healthcare system to potentially facilitate earlier treatment, identify groups at greater risk of diagnostic delay, and to help improve the overall diagnostic pathway.

This study protocol proposes a multicentre retrospective observational study of the UK and ROI MS diagnostic pathway. DIMES involves data collection direct from clinical records, by a unique, trained, and geographically diverse network of student and junior doctor collaborators, to provide new insights that cannot be provided by contemporary databases.

### Standard diagnostic service and relevant quality standards

There is no single guideline that provides a set diagnostic pathway for MS. National bodies, such as the National Institute for Health and Care Excellence (NICE), provide some guidance as to recommended standards in England. Recent position papers have employed the iterative Delphi method to develop benchmark standards, but no single position paper is considered the gold-standard in the UK [29–31].

Through reviewing guidelines (listed in Supplement S1), position papers, and proposed standards, and consulting a geographically diverse range of UK MS specialists, we developed the data dictionary (Supplement S2). Given the diagnostic journey of people with multiple sclerosis exhibits significant inter-person heterogeneity, the data dictionary allows for collection of data that deviates from a ‘typical’ diagnostic journey.

### Objectives

**Fig. 1:**
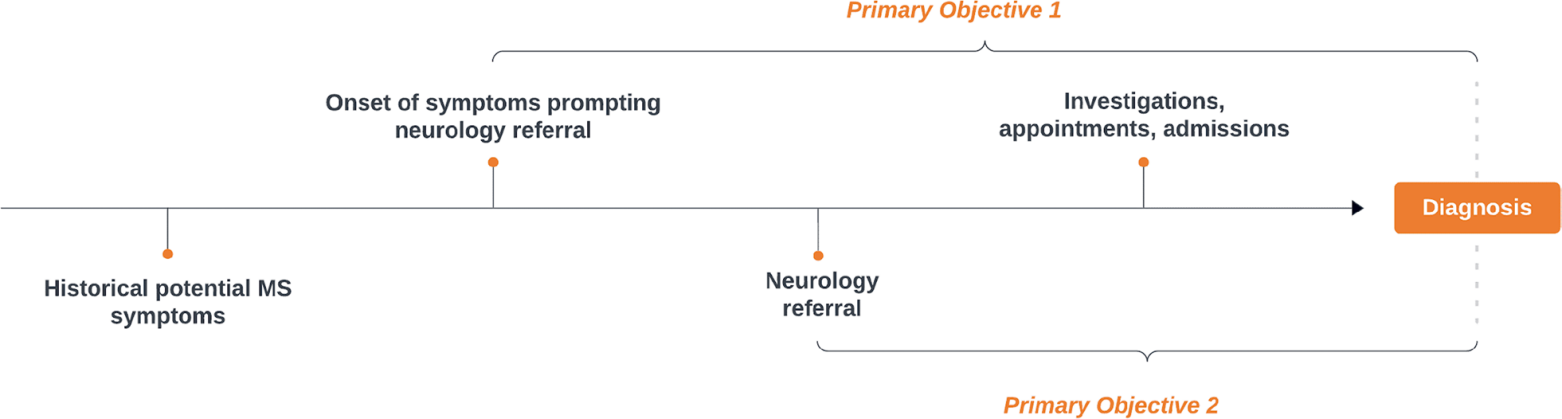
Primary objectives represented diagrammatically

The primary objective is to quantify the time taken for people to be diagnosed with MS in the UK and ROI using 1) time from symptoms/signs prompting referral to neurology (as listed in referral letter or neurology clinic letters), to MS diagnosis; and 2) time from the referral to neurology for suspected MS, to MS diagnosis (Fig. 1).

The secondary objectives are:

- To characterise the diagnostic journey of people with MS in the UK and ROI, including initial/presenting symptoms, referring specialties, investigations performed, time to completion of investigations, number of neurology appointments, functional status at diagnosis.
- To identify factors associated with diagnostic delay in the UK and ROI.
- To characterise the treatments prescribed for people with MS, including the use of DMTs and time to receipt.
- To evaluate the immediate post-diagnosis MS quality of care pathways in the UK by assessing adherence to NICE Quality Statement (QS) QS 108 Statements 1, 2 and 4, as outlined, verbatim, below [32].

### QS 108 guidelines

**Table:** 

Statement 1: Adults with multiple sclerosis (MS) are given support at the time of diagnosis to understand the condition, its progression and the ways it can be managed, by the consultant neurologist making the diagnosis.
*Audit Calculation: Proportion of adults with MS who are given information about MS, its progression and the ways it can be managed by the consultant neurologist at the time of diagnosis.*
Statement 2: Adults with multiple sclerosis (MS) are offered a face-to-face follow-up appointment with a healthcare professional with expertise in MS, to take place within 6 weeks of diagnosis.
*Audit Calculation: Proportion of adults with MS who have a face-to-face follow-up appointment with a healthcare professional with expertise in MS within 6 weeks of diagnosis.*
Statement 4: Adults with multiple sclerosis (MS) who have problems with mobility or fatigue are offered support to remain physically active.
*Audit Calculation: Proportion of adults with MS who are offered support to remain physically active.*

## Methods

### Study design

This is a retrospective, multicentre observational study, across centres providing MS diagnostic services in the UK and ROI. This study employs data collection directly from clinical notes, via a network of geographically diverse and trained student and junior doctor collaborators - NANSIG [33]. NANSIG is a student and junior doctor-led collaborative aiming to provide medical students and junior doctors with experience in neurology and neurosurgery, and has a regional lead network of university-affiliated medical students, and hospital-affiliated junior doctors.

### Centre eligibility

DIMES is open to any hospital in the UK or ROI that provides an MS diagnostic service. We expect DIMES will primarily enrol centres from large/teaching hospitals in the UK and ROI, due to the existing NANSIG regional lead network. In England, these will mainly be centres identified as N1/N2 in the Getting It Right First Time (GIRFT) report [34]. We aim to enrol ≥ 20 centres.

### Patient eligibility criteria

#### Inclusion criteria

We will include individuals who received a new diagnosis of MS between 1st July 2022 and 31st December 2022. This 6-month time period was selected to 1) balance data collection burden with the number of included individuals, 2) examine a period sufficiently outside the most critical influence of the COVID-19 pandemic, and 3) enable collection of post-diagnosis follow up data relating to use of DMTs and care.

Formal diagnosis by a clinician working in neurology should be clearly documented in the individual’s notes. In order to maximise case ascertainment, we will include individuals irrespective of investigations or treatments received, or the completeness/accessibility of their clinical data.

Each centre will recruit all consecutively diagnosed individuals in the specified time period. Individuals must have received their formal diagnosis (as evidenced by a letter/clinical note) from the centre, and not had their care transferred *into* the centre post-diagnosis (e.g., for DMT treatment). Individuals transferred *out* of the centre following diagnosis (e.g., for treatment at a tertiary centre) will remain eligible for inclusion.

#### Exclusion criteria

- We will exclude individuals with only a possible/suspected diagnosis of MS (as opposed to confirmed).

The number of people excluded following screening will be reported, including the rationale for exclusion.

### Patient identification

Local teams will work with a supervising consultant at participating centres to identify all individuals meeting inclusion criteria. Additional assistance may be provided by an MS Nurse or junior doctor, and the contributions of these individuals will be acknowledged in the study.

Suggested methods for identification in ordered preference include:

- 1^st^ line: Review of locally curated lists of individuals with MS, or new diagnoses.
- 2^nd^ line: Review of MS clinic appointments.
- 3^rd^ line: Review of general neurology / neurology clinic appointments.
- 4^th^ line: Review of individuals with relevant diagnostic hospital (ICD-10) or General Practice (Read Clinical Terms Version 3) code(s) (full list in Supplement S3).

Teams will be expected to report the method used for patient identification, with one method alone being sufficient. Methods based on use of MS-associated medications such as attendance at DMT infusion centres, Blueteq records or pharmacy records are not acceptable due to selection bias. Other methods of identification such as search of radiology databases for MS-related terms may be acceptable if discussed with the Steering Committee.

### Recorded variables

All measures, exposures and outcomes are defined in detail, in our data dictionary (Supplement S2), including the likely sources of information from the electronic health record.

#### Baseline measures and exposures

Demographic variables include: age; sex; ethnicity; index of deprivation (UK centres: index of deprivation decile and rank; ROI centres: Pobal Haase Pratschke (HP) Index 2016 and description); distance from home postcode to MS centre; employment status; and smoking status.

MS variables include: family history of MS (with the degree of relatedness of relative with MS); possible prior misdiagnosis or MS mimic; date of ‘First Relevant’ referral to neurology/MS clinic and source of this referral; symptoms/signs/abnormal investigations prompting this ‘First Relevant’ referral to neurology and date of onset; historical MS-associated symptoms/signs/abnormal investigations that precede ‘First Relevant’ referral to neurology and date of onset.

Neurology appointment and investigation variables include: date of first neurology appointment after referral (if available), and whether this neurologist had a practice focus in MS; documented follow up plan from this appointment; date of subsequent neurology appointment(s); date MRI requested, performed, and reported; MRI findings, region, use of contrast, and modality; date lumbar puncture requested, and performed; and lumbar puncture findings.

MS diagnosis variables include: date of diagnosis and whether the diagnosing neurologist had a practice focus in MS; previous diagnosis of CIS; MS subtype; number of MS relapses at the time of diagnosis; and disability scores at diagnosis (EDSS, Timed 25-Foot Walk Time, Nine-Hole Peg Test Time at Diagnosis (right and left hands), Fatigue Severity Score, and Multiple Sclerosis Functional Composite Score).

MS treatment variables include: DMT(s) received; date of referral for DMT MDT; date of DMT MDT; date of first DMT started; administration method.

Quality measures from QS 108 include: Support at diagnosis (including specifically support to (a) understand the condition, (b) understand MS progression and (c) understand MS management); Follow up after diagnosis; and Physical activity advice.

### Outcomes

#### Primary outcomes

The primary outcomes relate to time to diagnosis and include: 1) time from symptoms/signs prompting referral to neurology (as listed in referral letter or neurology clinic letter), to MS diagnosis; and 2) time from referral to neurology for suspected MS, to MS diagnosis.

These outcomes have been selected with equal weighting of importance, as the former may be most important from a patient perspective (as disease starts, from their perspective, when they first notice symptoms), whereas the latter gives a more meaningful evaluation of the diagnostic service.

#### Secondary outcomes

The outcomes relating to diagnostic journey include: a) symptoms prompting referral and earliest symptoms potentially relating to MS; b) which specialties refer to neurology for diagnostic work up for MS; c) the number and results of lumbar puncture and MRI investigations performed; d) the time from request of investigation to completion and to reporting; e) the number of neurology appointments between referral for suspected MS and MS diagnosis; and f) functional status at time of diagnosis (according to various scoring systems).

The outcomes relating to MS care include: a) the prescription of DMTs including the specific agents used and time to receipt after diagnosis; and b) adherence to QS 108 Statements 1, 2 and 4 (NICE Quality Statements, 2016).

### Data collection

**Fig. 2:**
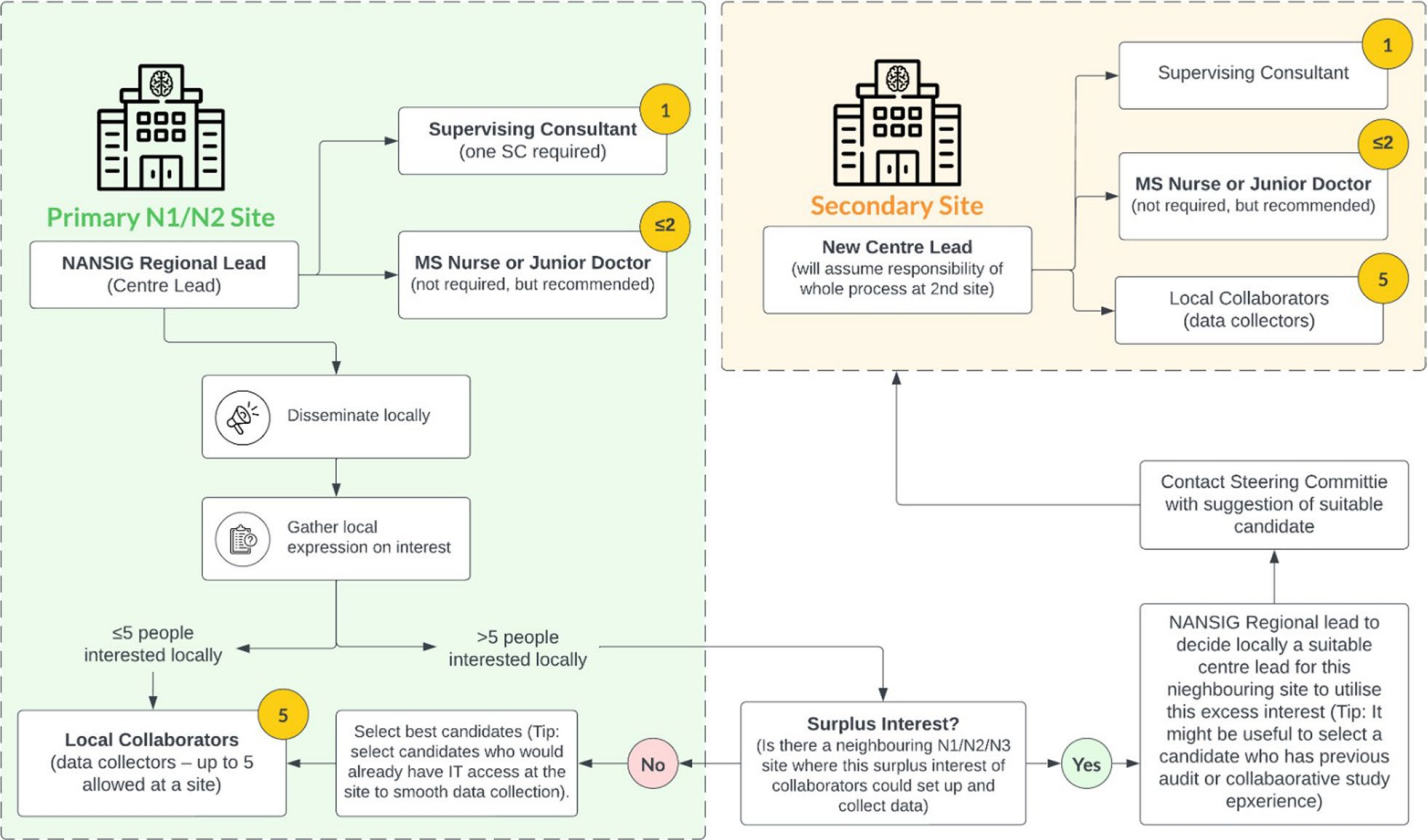
Centre team structure

At each centre, a team of collaborators will lead the data collection with guidance from a supervising consultant, and mandatory specific training from the study leads (Fig. 2).

Data collectors will extract information from existing clinical records onto a paper proforma (case report form (CRF)). The template for this proforma will be provided by the study steering team. These paper records will not leave hospital premises, and must be kept securely on site in a locker cabinet or similar, in accordance with Good Clinical Practice [35].

Following paper data collection, collaborators will enter data onto a standardised Microsoft Excel spreadsheet on password-protected NHS computers/servers (Supplement S4). Each new individual will be assigned a ‘Record ID’ [e.g. sequentially 1–100] on the spreadsheet. For the purposes of local traceability, a separate spreadsheet will be securely kept, matching each ‘Record ID’ to local ‘Hospital Number’. This separate spreadsheet contains identifiable information, is solely for internal use at the hospital centre, and must not be shared outside of the local collaborator team. This identifiable information should be deleted at final study completion (defined as post-data validation). CRFs should be disposed of appropriately, as per local hospital protocol.

Data will be collated centrally for analysis. Individual centres will not be identified by name in study outputs, in relation to centre-level data.

This Microsoft Excel spreadsheet will be sent to the steering committee at the study-specific nhs.net email from an nhs.net (or local equivalent) email to maintain confidentiality and security of the data.

### Data validation & quality assurance

#### Design

This protocol has been conceived and written with the guidance of an expert multi-specialty advisory group.

#### Training

To ensure clear understanding of the study topic, inclusion criteria, primary outcomes, and the principles of data governance, all collaborators must attend a centralised induction and training session delivered by the DIMES steering committee. DIMES centre leads are encouraged to hold meetings with collaborating team(s) to address any concerns with local setup of the study and/or any issues with data collection.

#### Centre team structure

Medical students will take the lead in disseminating and delivering this study alongside junior doctors and/or MS nurse specialists. The centre team structure consists of: 1) a centre lead (predominantly NANSIG university or deanery representatives); 2) supervising consultant; 3) MS nurse (optional); 4) non-consultant grade doctor (recommended for additional local support); and 5) up to five additional collaborators (Fig. 2).

#### Data completeness and consistency

Following data collection, only data sets with > 95% data completeness will be accepted for pooled national analysis. Data completeness is defined as the proportion of Excel spreadsheet cells with an appropriate entry including “not reported/not documented”. For the purposes of this study, ‘missing data’ exclusively refers to a lack of data field completion, or lack of justification for failure to complete the data field. Records with > 5% missing data points will be excluded from the study. Data sets from each centre will be assessed for completeness (as defined above) and consistency (such as possible errors) by the central study team on submission, with data sets returned to address omissions or areas of concern.

#### Data accuracy

We will use inter-collaborator agreement to assess the accuracy of submitted data. For this aspect of validation, 33 data variables have been selected on account of their relevance to the study’s objectives and statistical analyses (Supplement S5).

The centre lead (or another suitable individual if centre lead is not available) will perform independent data extraction (i.e., blinded to existing data entries) for 10% of the submitted records. These records will be selected randomly by the steering committee (using a random number generator on Microsoft Excel). Records that have already been reviewed by the centre lead will be excluded from this assessment. Once independent data extraction is complete for these randomly selected records, the centre leads will return this file for review to the steering committee. Comparison to original submissions for assessment of inter-reviewer agreement will then be carried out. The target for inter-reviewer agreement is ≥ 90% of data items. If inter-reviewer agreement is below the 90% threshold, an additional randomly selected 10% of records will be assigned using the above method for assessment of inter-reviewer agreement. If inter-reviewer agreement remains below 90%, the centre lead will be asked to validate all the centre’s records accordingly. Failure to comply with this will mean data from the respective centre may be analysed separately, or excluded from certain analyses. Conflicts between the original and re-submitted data will be resolved by discussion between the validator and local team, with oversight from the steering committee.

### Information sources

Data collectors at participating centres will be expected to review all the below information sources, where available, to ensure appropriately comprehensive review.

- Hospital electronic records
- Neurology clinic letter(s)
- Referral letter to neurology clinic
- Formal diagnosis letter/clinical note
- Prescription record at MS DMT centre / pharmacy records
- Radiology records of request dates and scans performed

We do not expect data collectors to review/access General Practice records for further information.

### Centre details and practice

We will circulate a survey to each enrolled centre for completion by the supervising consultant.

Intended measures will relate to QS 108 quality measures and local centre infrastructure.

Quality measures may include: support at diagnosis; follow-up after diagnosis; the availability of a single point of contact for coordinated care; the availability of a multidisciplinary team with expertise in MS; local arrangements to support people with MS to remain physically active; local arrangements regarding treatment for those who have a relapse; and comprehensive annual review.

Local centre infrastructure measures may include: the centre’s NHS England GIRFT categorisation; presence of on-site MRI scanners; local MS guidelines; current waiting times for neurology outpatient appointments; and presence of a dedicated MS nurse/nursing team.

Individual centres will not be identified by name in study outputs in relation to centre-level data. The survey is intended to help further characterisation of the diagnostic pathway of MS in the UK and ROI, and hopefully expand the context of the study results.

### Sample size

DIMES intends to recruit all eligible individuals from enrolled centres. Due to the non-random nature of centre enrolment, this represents a convenience sample. There is no minimum effect size pertinent to our primary outcomes or their analysis. As a result, a sample size calculation has not been performed as this would be inappropriate; however, we have estimated the study sample size based on available literature. We have used data from England to inform these estimates, on account of availability of data (on MS incidence, and provision of neurology services), and the expectation most centres will be enrolled from England.

Based on the estimated 4,950 new MS diagnoses annually in England, we estimate that 2,475 diagnoses will occur during the 6-month study period [36]. For the below calculations we have assumed that MS diagnoses only occur at centres where neurologists are based (given the McDonald criteria requirement of diagnosis by a neurologist) [13].

- According to GIRFT, neurologists are based at 118 centres in NHS England, across 24 N1 (inpatient neurology & neurosurgery), 27 N2 (inpatient neurology only), and 67 N3 (neurologists are based, without inpatient neurology) centres [34].
- Assuming similar caseloads at N1, N2 and N3 centres, diagnoses at N1 and N2 centres may encompass 43% (number of N1 or N2 centres / number of N1, N2 or N3 centres, 51/118) of the total in England.
- 43% of 2,475, yields an estimate of 1064 diagnoses.
- As we aim to enrol ≥ 20 centres, of the existing 51 N1/N2 centres we estimate a minimum study population (39% of 1,064 diagnoses), i.e., 415.
- If we assume similar caseloads across the estimated 20 included centres, we anticipate ~21 individuals per centre.

### Statistical analysis

#### Descriptive analyses

For continuous variables with a normal distribution, mean and standard deviations (SDs) will be presented. Where continuous variables have skewed distributions, medians, 25th and 75th percentiles, or interquartile ranges will be presented. For categorical variables, summary statistics, frequency, and percentages of each category will be calculated.

Demographic and clinical characteristics of included individuals will be summarised in tables.

Adherence to quality standards will be summarised as percentages. Time-to-event variables (such as time from symptom onset to diagnosis, or time to starting DMT) will be presented descriptively with median and interquartile ranges, and graphically in the form of survival curves. For post-diagnosis variables (such as those relating to DMT), survival curves will be censored at the centre-level at the date of data collection completion. We do not intend to censor time to diagnosis or pre-diagnosis variables.

#### Multivariable analysis: factors associated with time to diagnosis

Provided the proportional hazards assumption is met, we will use Cox proportional hazards models to investigate the association of factors with time to MS diagnosis. We will conduct this multivariable analysis using each of our primary outcomes: 1) using time from symptoms/signs prompting referral to neurology, to MS diagnosis, as the outcome variable; and 2) using time from referral to neurology for suspected MS, to MS diagnosis.

We have selected variables for investigation from the existing literature of factors that are potentially associated with diagnostic delay of MS. These variables include: 1) demographic factors (age, sex, ethnicity, socioeconomic deprivation, distance from individual’s home postcode to MS centre, employment status, smoking status); 2) MS details (family history, possible previous misdiagnosis, symptoms, Expanded Disability Status Scale (EDSS) score); and 3.) investigations (time from MRI request to report, time from lumbar puncture request to report), with time to diagnosis of MS. As there is limited evidence of association of investigation variables with diagnostic delay, these should be considered exploratory.

We will exclude individuals with missing start, or event, dates in the above analyses.

#### Secondary analyses

We will explore the associations of socioeconomic deprivation with aspects of MS care using multivariate analyses. We will use the selected indices of deprivation as regional measures of deprivation. Aspects of MS care include: time to MS diagnosis (time from symptoms/signs prompting referral to neurology, and time from referral to neurology for suspected MS); number of, and time to investigations (MRI and lumbar punctures); measures of quality of MS care (QS 108 measures: support at diagnosis, follow up after diagnosis, physical activity); and prescription of DMTs.

If there is sufficient use of diagnostic codes for patient identification, we will present their positive predictive values.

## Discussion

### Study approval and registration

This study will not change local routine practice and will use data obtained as part of usual care. The UK Health Research Authority (HRA) decision tool has confirmed that this study is not considered research by the NHS and does not require NHS research ethics committee approval (Supplement S6) [37].

The centre lead and accompanying research team at each unit are responsible for registering the study with appropriate approvals, in line with national guidelines. In the UK, this will usually be as a service evaluation study. Supervision by a local neurology consultant is required for this process, therefore it is the responsibility of the local teams to identify suitable consultants for study registration. Collaborators will be required to confirm that a local approval is in place prior to data collection.

### Data governance

Data will be collected onto purpose-designed CRFs and entered into a bespoke Microsoft Excel spreadsheet at the respective centres’ password-protected hospital server. All data should be handled in accordance with local data governance policies, and all paper copies of any data should be destroyed as confidential waste within the centre, once uploaded onto the local hospital server. At the end of the data collection period, de-identified data will be transferred to the DIMES Steering Committee via a secure nhs.net encrypted email (or local equivalent), for data cleaning and analysis. For information governance, patient identifier data will be stored securely locally, but will not be sent on to the DIMES Steering Committee. No patient data will be transferred without prior local permissions.

Data will be collated, stored and analysed by the DIMES Steering Committee. Data collected during the DIMES study can be used for future analyses at the steering committee’s discretion.

### Protocol dissemination

The protocol will be disseminated primarily through recruited medical student collaborators in the NANSIG network. Should local neurology units wish to view the protocol, this will be freely accessible from the DIMES study hub on the NANSIG website (www.nansig.org). Any publications of the protocol will be advertised through social media.

### Dissemination of results

Following study completion, teleconferences will be held with all collaborators to share and discuss the data analysis undertaken and the study results. Following this, results will be presented at local, regional, national, and international conferences by collaborators. A standardised PowerPoint presentation and poster will be created for this purpose. All presentations will be coordinated by the DIMES steering committee to avoid duplications and to ensure all conference regulations are fulfilled. In addition, the results will be disseminated via publication in a peer-reviewed medical journal. The study results will be reported using the Strengthening the Reporting of Observational studies in Epidemiology (STROBE) guidelines [38].

Following publication, the manuscript can be shared by collaborators with their local neurology units to discuss the study results, and to highlight the scope for areas of improvement for MS diagnosis [39]. Local teams at each neurology unit can request for their respective centre data from the steering committee following study completion.

### Supplementary Information

**Supplementary Material 1.**

## Data Availability

No datasets were generated or analysed during the current study.

## Abbreviations

MS: Multiple Sclerosis
UK: United Kingdom
ROI: Republic of Ireland
DIMES: Delays in Multiple Sclerosis Diagnosis
NANSIG: The Neurology and Neurosurgery Interest Group
PPI: Patient and Public Involvement
DMT: Disease modifying therapy
MRI: Magnetic resonance imaging
COVID-19: Coronavirus Disease 2019
NICE: National Institutes for Health and Care Excellence
QS108: Quality Statement 108 (NICE)
GIRFT: Getting It Right First Time
ICD-10: International Classification of Diseases, Tenth Revision
EDSS: Expanded disability status scale
CRF: Case report form
NHS: National health service (UK)
HRA: Health Research Authority
STROBE: Strengthening the reporting of observational studies in epidemiology
