# Supplementary material for: Delays in Multiple Sclerosis diagnosis (DIMES): protocol for a multicentre, observational study of multiple sclerosis diagnostic pathways in the United Kingdom and Republic of Ireland
Source: BMC Neurol. 2024 Mar 28;24:105. doi: 10.1186/s12883-024-03598-z (PMC10976685; doi:10.1186/s12883-024-03598-z)
Supplement: Supplementary file 1 — Supplementary Material 1. [file 12883_2024_3598_MOESM1_ESM.docx]

**Supplement**

**S1: Guidelines reviewed to inform design of data dictionary**

- NICE guideline NG220 Multiple sclerosis in adults: management, published 22 June 2022.
- NICE guidelines on Ocrelizumab [TA533]; Beta interferons and glatiramer acetate [TA527]; Dimethyl fumarate [TA320]; Alemtuzumab [TA312]; Teriflunomide [TA303]; Fingolimod [TA254]; Ofatumumab [TA699]; and Natalizumab [TA127]. (Technology Appraisals)
- NICE Multiple sclerosis Quality standard (QS) 108, 2016.
- MS Brain Health Consensus Standards - Available from: https://www.msbrainhealth.org/healthcare-professionals/ms-brain-health-consensus-standards/
- Optimum Clinical Pathway: Multiple Sclerosis (Neural) - Available from: https://www.neural.org.uk/wp-content/uploads/2020/07/Optimum-pathway-for-patients-with-MS_updated.1.pdf

**S2: DIMES Data Dictionary: “Data Collection Guide for Collaborators”**

The data collection guide is available from this read-only Google Drive link:

[*https://docs.google.com/document/d/1cxDrdI2OJnAFE6T4aJJmruFvOaQpCO6_dQKMxBKz-ZU/edit?usp=sharing*](https://docs.google.com/document/d/1cxDrdI2OJnAFE6T4aJJmruFvOaQpCO6_dQKMxBKz-ZU/edit?usp=sharing)

**S3: Diagnostic codes list**

**ICD-10 code for MS (Inpatient/hospital coding systems):**

G35 Multiple sclerosis

**Read Clinical Terms Version 3 code for MS (General Practice coding systems):**

F20..00 Multiple sclerosis

F20..11 Disseminated sclerosis

F200.00 Multiple sclerosis of the brainstem

F201.00 Multiple sclerosis of the spinal cord

F202.00 Generalised multiple sclerosis

F203.00 Exacerbation of multiple sclerosis

F204.00 Benign multiple sclerosis

F205.00 Malignant multiple sclerosis

F206.00 Primary progressive multiple sclerosis

F207.00 Relapsing and remitting multiple sclerosis

F208.00 Secondary progressive multiple sclerosis

F20z.00 Multiple sclerosis NOS

**S4: Microsoft Excel Data Collection Tool**

The Microsoft Excel Data Collection Tool is available from this read-only Google Drive link:

[*https://docs.google.com/spreadsheets/d/1jMV7wNTZgkVpfAyUBJjcKZekrozE5NYT/edit?usp=sharing&ouid=116265156228735083082&rtpof=true&sd=true*](https://docs.google.com/spreadsheets/d/1jMV7wNTZgkVpfAyUBJjcKZekrozE5NYT/edit?usp=sharing&ouid=116265156228735083082&rtpof=true&sd=true)

**S5: Data validation checks Microsoft Excel spreadsheet**

The Microsoft Excel Data Validation Spreadsheet is available from this read-only Google Drive link:

[*https://docs.google.com/spreadsheets/d/12w3iounsicAcc5cMPc0p-9HqZKzcvChCnDbdmuU8FaA/edit?usp=sharing*](https://docs.google.com/spreadsheets/d/12w3iounsicAcc5cMPc0p-9HqZKzcvChCnDbdmuU8FaA/edit?usp=sharing)

**S6: Health Research Authority and NHS Research Ethics Committee assessment tools**


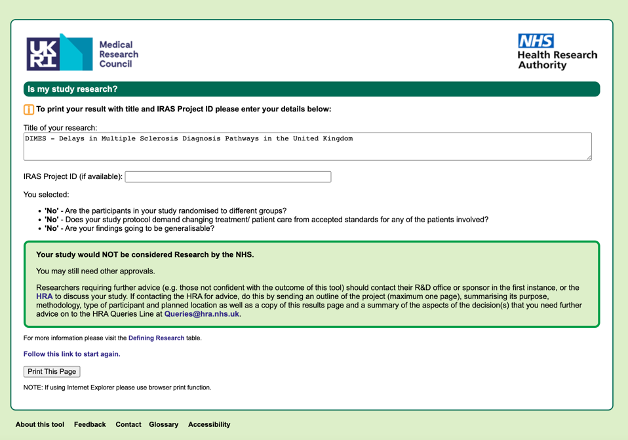


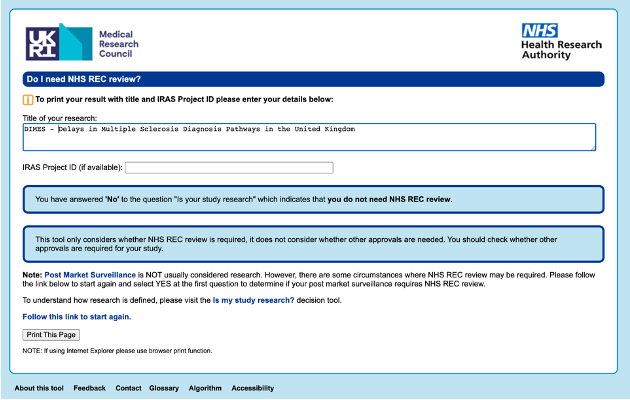


**S7: Authorship eligibility**

In accordance with National Research Collaborative (NRC) authorship guidelines, all research outputs from DIMES will be listed under a single corporate authorship model: ‘Neurology and Neurosurgery Interest Group (NANSIG)’ (34). All collaborators will be listed as PubMed-citable collaborators in accordance with the roles defined below upon demonstration of satisfactory completion of the minimum requirements for authorship.

- Writing and Data Analysis Group: A group of medical students, junior doctors and external advisory board members responsible for the overall scientific content, data analysis, and preparation of manuscripts.
- Steering Committee: A core group of medical students and junior doctors who have overall responsibility for protocol design, project coordination, and data handling.
- Centre leads: The main point of contact for data collection at each centre, and is responsible for local site setup and governance. Centre leads should be identified and recruited by NANSIG and are recommended to be a junior doctor or senior medical student within the local hospital team. Only one individual can fulfil the role of centre lead per local team. Requirements for authorship on DIMES outputs include:
  - Primary person responsible in obtaining local approvals for conduct of DIMES (e.g. registration, seeking Caldicott guardian permission and information governance approval, as required).
  - Active involvement in a local team during a data collection period at a centre which meets the criteria for enrolment.
  - Presentation of local DIMES results at their centre (or arranges another collaborator to present on their behalf).
- Local collaborators (data collectors): In addition to the centre lead, up to five collaborators may be responsible for data collection at a particular centre. The individuals will most commonly be medical students. Minimum requirements for authorship on DIMES outputs include:
  - Compliance with local approval processes and data governance policies.
  - Active involvement in data collection
  - Collaboration with the centre lead to ensure that the audit results are reported back to the audit office / clinical teams.
- Junior doctor(s) and/or MS nurse specialist(s): Local teams may also include a junior doctor(s) or an MS nurse specialist(s) (max 2 total) to supervise/support local. teams. Their roles include:
  - Providing advice around clinical details and their diagnosis
  - Liaising with multidisciplinary teams to aid complete case ascertainment
- Supervising Consultant: Data collection in each hospital must be supervised by a consultant. Minimum requirements for authorship on DIMES outputs include:
  - Sponsorship of local registration, and responsible to ensure local collaborators act in accordance with local governance guidelines.
  - Facilitation of local presentation and consideration of appropriate post-audit interventions/improvements after DIMES.
  - Completion of DIMES Centre Details and Practice survey.
  - Completion of workplace-based assessments for students or trainees (ePortfolio), if requested.
